# Prevalence and intensity of soil-transmitted helminth infections among school-aged children in five districts in Uganda

**DOI:** 10.1371/journal.pntd.0012324

**Published:** 2024-08-01

**Authors:** Benjamin Tinkitina, Prudence Beinamaryo, Hilda Kyarisiima, Betty Nabatte, Moses Arinaitwe, Alfred Mubangizi, Paul Emerson, Kristin M. Sullivan

**Affiliations:** 1 Vector Borne and Neglected Tropical Diseases Division, Ministry of Health, Kampala, Uganda; 2 Children Without Worms, The Task Force for Global Health, Decatur, Georgia, United States of America; Consejo Nacional de Investigaciones Cientificas y Tecnicas, Fundación Mundo Sano, ARGENTINA

## Abstract

**Background:**

Soil-transmitted helminth (STH) infections, commonly caused by roundworms (*Ascaris lumbricoides*), whipworms (*Trichuris trichiura*), and hookworms (*Necator americanus* and *Ancylostoma duodenale*), were widespread among Ugandan schoolchildren in the late 1990s and early 2000s. Since 2003, the Ugandan Ministry of Health has administered biannual preventive chemotherapy to children aged 1–14 years to control these infections. Twenty years after the program’s inception, there is scant data to show the long-term impact of these national deworming efforts.

**Methods:**

To estimate the prevalence and intensity of STH infections among 10–14-year-old primary school children, school-based, cross-sectional surveys were conducted in November 2023 across five districts (Kamwenge, Sheema, Adjumani, Lamwo, and Zombo). Sixty-five children from five schools per district were selected for inclusion. Fecal egg counts were determined using the Kato-Katz microscopy technique, performed in duplicate by trained laboratory technicians.

**Results:**

The survey findings revealed a high prevalence of any STH infection in Kamwenge District (21.2%, 95% confidence limits (CL): 5.7%, 36.6%), while the remaining four districts exhibited lower prevalences, ranging from 0.4% (95% CL: 0.0%, 1.2%) in Adjumani District to 5.6% (95% CL: 0.0%, 11.4%) in Sheema District. The prevalence of moderate-to-heavy-intensity infections was below 1% across all districts. *A*. *lumbricoides* was identified infrequently. Hookworm infections were primarily identified in the western districts of Kamwenge and Sheema, while *T*. *trichiura* infections were common only in Kamwenge District. Hookworm and *T*. *trichiura* infections were uncommon in the northern districts of Adjumani, Lamwo, and Zombo.

**Conclusions:**

These surveys suggest that morbidity due to STH infections among schoolchildren may be well controlled in these five districts, as evidenced by low moderate-to-heavy-intensity infection prevalence. However, the prevalence of any intensity infection remains elevated in some districts, indicating the need for continued preventive chemotherapy distribution. A reduction from biannual treatment may be warranted in four districts, per World Health Organization recommendations.

## Author summary

Intestinal worm infections, also known as soil-transmitted helminth (STH) infections, are a significant public health concern that can impair physical growth, lead to anemia, and affect cognitive development among children. Initial surveys in Uganda between 1998 and 2005 showed a high prevalence of STH infections, with over 50% of school-aged children affected in many districts. In response, the Ugandan Ministry of Health initiated biannual deworming campaigns targeting school-aged children, along with health education programs.

Our follow-up surveys in November 2023 across five districts aimed to evaluate the impact of these deworming campaigns on the prevalence of STH infections. We found a substantial decrease in STH prevalence in four districts, suggesting that the national deworming program has been effective and could potentially reduce the frequency of deworming campaigns in these areas. However, in Kamwenge District, the STH prevalence remained elevated, indicating a need for further investigation to understand the persistence of infections and to enhance control measures to meet World Health Organization targets.

## Introduction

Soil-transmitted helminth (STH) infections are caused by roundworms (*Ascaris lumbricoides*), whipworms (*Trichuris trichiura*), and hookworms (*Necator americanus* and *Ancylostoma duodenale*). The burden of soil-transmitted helminthiases is substantial, and STH are among the most common infections globally, with an estimated 1.5 billion people affected [1]. These infections represent a group of neglected tropical diseases that disproportionately affect impoverished communities in developing countries and are often found in populations with inadequate sanitation and poor hygiene practices [2,3].

Control efforts are primarily centered around the mass distribution of preventive chemotherapy (PC), a critical approach to reducing the prevalence and intensity of STH infections. Between 2008 and 2018, over 3.3 billion benzimidazole tablets (albendazole or mebendazole) were given to school-aged children, achieving coverage of >60% [1,4]. However, varying levels of success across different regions have been observed [5,6]. In India, for example, the prevalence of STH infections in some states was dramatically reduced after several rounds of mass drug administration, reaching a level below the World Health Organization (WHO) threshold for mass distribution [7–9]. On the contrary, despite significant efforts in Ethiopia, the prevalence of STH infections among school-age children remains high, indicating an ongoing challenge in achieving STH goals in the country [10]. These findings suggest that, while deworming and mass treatment campaigns may effectively reduce the burden of STH infections, achieving substantial and sustained reductions in prevalence requires integrated approaches that may include enhanced water, sanitation, and hygiene interventions, improved diagnostics, and targeted treatment strategies [2,3,11].

In Uganda, baseline surveys conducted between 1998 and 2005 revealed a high prevalence of STH infections, exceeding 50% among school-aged children in most districts surveyed [12,13]. The distribution of these infections across the country was uneven, with hookworms being widespread, while *A*. *lumbricoides* and *T*. *trichiura* showed higher prevalence in the southwest and were largely absent in the arid northeast. In response to these findings, the Ugandan Ministry of Health embarked on a national control strategy in 2003, adhering to WHO guidelines for PC treatment [5]. This initiative initially focused on areas where schistosomiasis annual mass treatment campaigns were being conducted. However, it was later expanded nationwide through integrated ‘Child Health Days,’ where children ages 1–14 years are treated biannually in schools and outreach posts.

Despite these efforts, there have been challenges in assessing the national impact of the deworming program due to limited evidence, particularly in areas where schistosomiasis is nonendemic. In addition, recent community-based surveys in five districts in Uganda indicated a marked decrease in STH prevalence in three of the five surveyed districts, suggesting the impact of long-term deworming is heterogeneous in the country [14]. District-specific evidence is required to optimally guide STH control programming throughout the country. Because of these challenges, the national control program has not significantly altered its treatment strategy since its inception nearly twenty years ago.

Northern and mid-western Uganda currently lack STH-specific impact surveys and have limited evidence available regarding program monitoring. Because of these gaps, the Vector Borne and Neglected Tropical Diseases Division prioritized these areas for epidemiological surveys to gather more detailed and geographically expansive evidence to inform programmatic decision-making and to potentially reduce program delivery costs by tailoring PC rounds to the current epidemiological situation.

### Objectives

These school-based surveys aimed to estimate the prevalence and intensity of STH infections among children aged 10–14 years in five districts in Uganda.

## Methods

### Ethics statement

The survey protocol received ethical approval from the Vector Control Division Research Ethics Committee (VCDREC153/4) and the Uganda National Council of Science and Technology (HS1937ES). The authorities of each participating district and school granted permissions. The consent process involved securing written consent from the parents or guardians of student participants. Additionally, each student was engaged in an assent process to confirm their understanding and willingness to participate in the survey.

### Population and setting

School-based surveys enrolling children aged 10 to 14 years were conducted in November 2023. This age group was selected per WHO guidelines [15] and is recommended because infection prevalence is expected to be highest among these children. Selected schools were asked to plan PC distributions after the survey to ensure that school-based deworming had not been performed since the last PC round (roughly six months prior).

**Fig 1:**
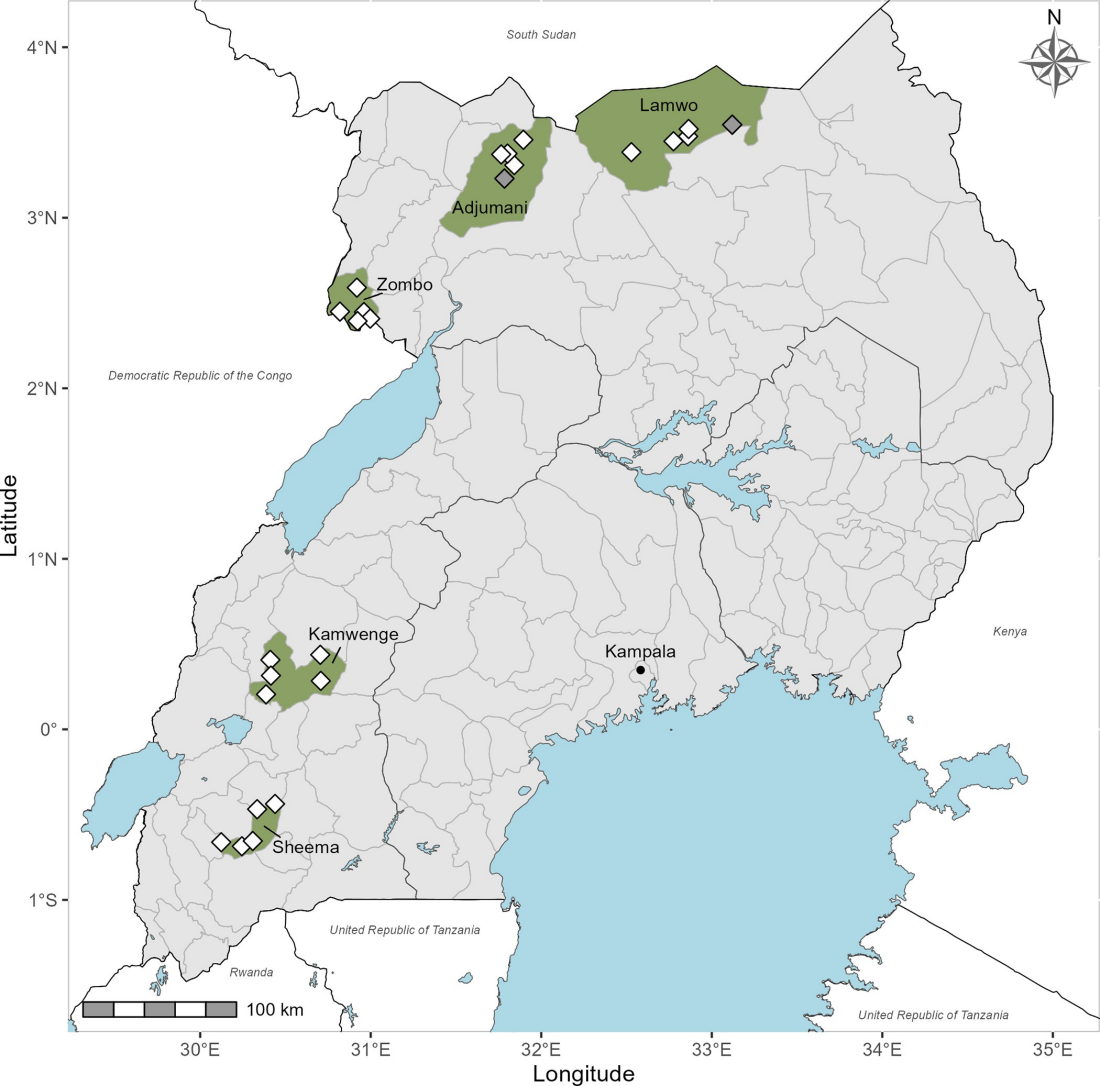
Districts surveyed to assess soil-transmitted helminth infection prevalence during November 2023. White diamonds represent the approximate location of the 23 surveyed schools; gray diamonds represent the approximate location of the 2 schools surveyed, but later excluded. The map was prepared using R software using basemap from ESPEN (2022) Admin 0, 1, and IU shapefiles (retrieved from https://espen.afro.who.int/tools-resources/data-query-tools/cartography-database on 12Dec2023) and The World Bank (2018) Africa Water Bodies shapefile (retrieved from https://datacatalog.worldbank.org/search/dataset/0040797 on 30May2022).

The Ministry of Health aimed to select five districts for surveys based on three considerations. First, they targeted districts lacking previous STH surveys. Second, they prioritized districts of programmatic interest. Last, district selection was based on preliminary geospatial modeling, conducted by colleagues at the Swiss Tropical and Public Health Institute, that identified locations where collected data could provide valuable insights for future STH prevalence prediction models. Based on this rationale, Sheema and Kamwenge Districts were selected from the Western region, and Adjumani, Lamwo, and Zombo from the Northern region (Fig 1).

### Mass drug administration

All selected districts have implemented biannual deworming programs for children aged 1–14 years through school and community outreaches by health workers. Per ESPEN (https://espen.afro.who.int/countries/uganda), from 2018–2022, effective (≥75%) program coverage was achieved during all years in Kamwenge, Adjumani, and Lamwo, while effective coverage was achieved in two out of five years in Sheema and Zombo.

Kamwenge District, also burdened with podoconiosis due to volcanic soils, eliminated onchocerciasis in 2016 and now relies on Child Health Days for STH treatment. Sheema District is also STH endemic and utilizes Child Health Days to treat children through school or outreach programs. Adjumani, Lamwo, and Zombo districts are co-endemic with schistosomiasis, lymphatic filariasis, and onchocerciasis. These three districts stopped mass drug administration for lymphatic filariasis with ivermectin and albendazole in 2017. Treatment for onchocerciasis ceased in Zombo in 2018, whereas Adjumani and Lamwo Districts still conduct mass drug administration using ivermectin (provided to all children and adults ≥5 years old). Lamwo District conducts district-wide treatment for onchocerciasis, while Adjumani District limits its distribution to specific sub-counties.

### Environment

The western districts of Sheema and Kamwenge share similarities in their tropical climate, bimodal rainfall patterns, and diverse vegetation. However, Sheema is smaller in size with an average annual rainfall of approximately 350 mm, while Kamwenge covers a larger area and experiences higher rainfall ranging from approximately 1,000 mm to 1,400 mm annually [16–18]. Lamwo, Adjumani, and Zombo Districts each present distinct geographical and climatic features. Lamwo, the largest district, experiences a tropical climate with bimodal rainfall, predominantly woody savannah vegetation, and an approximate annual rainfall of 1,300–1,500 mm [19]. Adjumani also has moderate bimodal rainfall, with a variety of ecosystems including savannah woodlands, seasonal swamps, and equatorial forest, with an annual rainfall ranging from 750 mm to 1,500 mm [20]. Zombo District has a tropical wet and dry climate with notably lower rainfall (an annual average of approximately 230 mm) and is characterized by savannah grasslands and scattered pockets of forests [21].

### Survey methodology

#### Design

We used a two-stage cluster sampling design in each of the five districts. In the first stage, we selected five schools in each district from a list of all public and private schools with 10–14-year-olds in attendance (Kamwenge n = 84 schools; Sheema: n = 82; Adjumani: n = 114; Lamwo: n = 104; Zombo: n = 86). Schools were randomly selected proportional to their student body size, using results from the latest primary enrollment figures.

In the second stage, 65 students aged 10–14 years at each school were selected using systematic sampling during the survey. The sampling interval was calculated as the number of 10–14-year-olds in line at the school divided by 65 and rounded up to a whole number. If the end of the line was reached, the sampling continued at the front of the line. Based on the survey design, each student 10–14 years old in the district had an equal probability of selection to the extent possible.

The school and student sample size per district adhered to the *WHO Guide for Mapping Neglected Tropical Diseases Amenable to Preventive Chemotherapy in the African Region* **[16]**. We surveyed an extra 15 students beyond the recommended 50 to account for potential non-enrollment or the inability of students to provide stool samples.

#### Community sensitization and consent

In the days before the survey, a Ministry of Health officer visited the district and schools for mobilization and sensitization. In each district, the District Health Team, political leaders, and religious leaders supported efforts to sensitize and mobilize the community.

Identifying eligible participants began a day before the survey, utilizing the school enrollment register to identify age-eligible students. These students were organized in a line and 65 students were selected. The selected students were asked to bring their parents the next day for the survey.

On the survey day, teachers and surveyors led the consent process for the parents, providing them with detailed information about the survey’s purpose and answering any questions that arose. Parents who consented for their children signed written consent forms. Parents were given transport refunds to offset the costs incurred while coming for the consent process.

#### Conduct

On the survey day, the data manager and collaborating teachers worked with the survey teams to conduct the surveys at each school. One survey team was assigned to each school, consisting of a Ministry of Health-trained enumerator and four laboratory technicians. Each survey team surveyed two schools per day, thus completing surveys in the five schools of the district over three days.

The enumerator began by collecting school-related information from the head teacher or his/her designee. After consenting to the survey, the parents of enrolled students were interviewed alongside their children to gather information on the child’s deworming history and handwashing practices. Each child was then assigned a unique QR code for identification. The enrolled students were then given thorough instructions on the stool collection process. Each student received a collection kit that included a stool container, old newspaper, and toilet paper for the sample collection. To ensure accurate identification, each stool container was marked with the QR code that matched the child’s unique QR code.

### Outcome assessment

#### Laboratory assessment

The laboratory team of trained parasitologists set up a field laboratory on the school premises. Samples were processed immediately upon their arrival. Per WHO guidelines, double-slide Kato-Katz microscopy was used to quantify the number of eggs in the stool sample, [22] and slides were read within 60 minutes of preparation [23]. Microscopists used a paper-based form to record the slide (A or B), the unique identifier of the stool sample, and the egg count per slide for hookworm, *A*. *lumbricoides*, and *T*. *trichiura*. The information collected on the paper-based form was later entered into an ODK Collect (https://getodk.org/) form for digital record-keeping.

#### Classification of infection intensity

The number of eggs per gram (epg) of stool was calculated by multiplying the average of the eggs per slide of the two slides by 24 (standard calculation based on 41.7 mg of stool per Kato-Katz smear preparation). In the few instances where only one slide reading was available, the single slide reading was used and multiplied by 24.

The epg estimate was used to classify the samples by infection intensity per WHO guidelines [23]. For *A*. *lumbricoides* infections, light intensity was defined as 1–4,999 epg, moderate intensity as 5,000–49,999 epg, and heavy intensity as ≥50,000 epg. *T*. *trichiura* infections were classified as light intensity with 1–999 epg, moderate intensity with 1,000–9,999 epg, and heavy intensity with ≥10,000 epg. For hookworm infections, light intensity was defined as 1–1,999 epg, moderate intensity as 2,000–3,999 epg, and heavy intensity as ≥4,000 epg.

### Data collection and management

Electronic ODK survey forms were used to collect school information, student demographics, and laboratory specimen results. Data were stored locally on smartphones and uploaded to a cloud-based server when a connection became available. The forms were linked using the individual’s unique code. The data managers conducted daily checks for data quality, consistency, and cleaning of submitted data.

### Statistical analyses and software

Within each district, we calculated prevalence estimates for hookworm, *A*. *lumbricoides*, and *T*. *trichiura*; we also estimated the prevalence of infection caused by any of these species. These estimates were calculated for two categories: infections of any intensity and infections of moderate-to-high intensity, resulting in eight prevalence estimates per district. We calculated the design-corrected 95% confidence limits for each estimate. Data management, cleaning, and analyses were conducted using R version 4.3.2.

## Results

### Survey population

Table 1 shows the number of schools and students surveyed and select demographics of survey participants. Five schools were surveyed in each district. However, it was later learned that two schools (one in Adjumani District and one in Lamwo District) had conducted deworming within the month prior. These two schools were excluded from the analysis (see S1 Text for further information).

**Table 1:** Characteristics of surveyed schools and students by district.

District	Kamwenge	Sheema	Adjumani	Lamwo	Zombo
Region	Western	Western	Northern	Northern	Northern
**Schools**					
Surveyed (n)	5	5	4	4	5
WaSH factors (n (%*))					
Has latrine					
No	0 (0%)	0 (0%)	0 (0%)	0 (0%)	0 (0%)
Yes	5 (100%)	5 (100%)	4 (100%)	4 (100%)	5 (100%)
Has hand-washing facility					
No	0 (0%)	0 (0%)	0 (0%)	0 (0%)	0 (0%)
Yes, with water only	3 (60%)	4 (80%)	0 (0%)	2 (50%)	2 (40%)
Yes, with water and soap	2 (40%)	1 (20%)	4 (100%)	2 (50%)	3 (60%)
Improved status of primary source of water^†^					
Unimproved	0 (0%)	0 (0%)	0 (0%)	0 (0%)	0 (0%)
Improved	5 (100%)	5 (100%)	4 (100%)	4 (100%)	5 (100%)
**Students**					
Surveyed (n)	307	303	244	245	299
Class range	1–6	1–6	2–6	1–5	1–6
Male	47.6%	52.5%	42.4%	47.2%	47.2%
Mean age (years)	11.4	11.4	11.8	11.6	11.3
History/observations (n (%*))					
Ever swallowed deworming medication (reported)					
No	8 (2.6%)	68 (22.4%)	1 (0.4%)	3 (1.2%)	4 (1.3%)
Yes	299 (97.4%)	235 (77.6%)	242 (99.6%)	240 (98.8%)	295 (98.7%)
Unknown	0	0	1	2	0
Usually washes hands (reported)					
No	37 (12.1%)	29 (9.6%)	19 (7.9%)	2 (0.8%)	87 (29.2%)
Yes, without soap	177 (57.8%)	181 (59.7%)	162 (67.5%)	215 (88.5%)	181 (60.7%)
Yes, with soap	92 (30.1%)	93 (30.7%)	59 (24.6%)	26 (10.7%)	30 (10.1%)
Unknown	1	0	4	2	1
Type of shoes worn (observed)					
None	51 (16.6%)	108 (35.6%)	35 (14.3%)	99 (40.7%)	70 (23.4%)
Open-toed	196 (63.8%)	165 (54.5%)	178 (73.0%)	132 (54.3%)	208 (69.6%)
Closed-toed	60 (19.5%)	30 (9.9%)	31 (12.7%)	12 (4.9%)	21 (7.0%)
Unknown	0	0	0	2	0

Abbreviations: n–number; WaSH–water, sanitation, and hygiene

^*^All school responses are known; for students, *percentages do not include unknown responses in the denominator*. *Decimal places deliberately differ between schools and students*.

^*†*^*Defined as improved/unimproved per WHO/UNICEF Joint Monitoring Programme classifications for drinking water; improved drinking water sources include*: *piped water*, *boreholes or tubewells*, *protected dug wells*, *protected springs*, *rainwater*, *and packaged or delivered water*. *(Source*: *https*:*//washdata*.*org/monitoring/drinking-water**)*

On average, approximately 60 students per school provided stool samples in each district. The mean age of surveyed students ranged from 11.3 to 11.8 years within the districts. While the sex distribution was roughly equal in most districts, in Adjumani District, the percentage of male students (42.4%) was notably lower compared to the other surveyed districts.

All 23 schools reported having latrines and improved water sources. While hand-washing facilities with water were available in all schools, access to soap was limited, with only approximately half of the schools reporting its availability. In four districts, nearly all students (>97%) reported having ever swallowed deworming medications, while in Sheema District, the proportion was lower, with only about 75% reporting it. In Zombo District, about 70% of students reported that they usually wash their hands, while in other districts this proportion was higher (≥85%). Interviewer-observed shoe use varied across districts, ranging from lows of 59.3% and 64.4% in Lamwo and Sheema Districts, respectively, to a high of 85.7% in Adjumani District.

### Prevalence estimates

**Fig 2:**
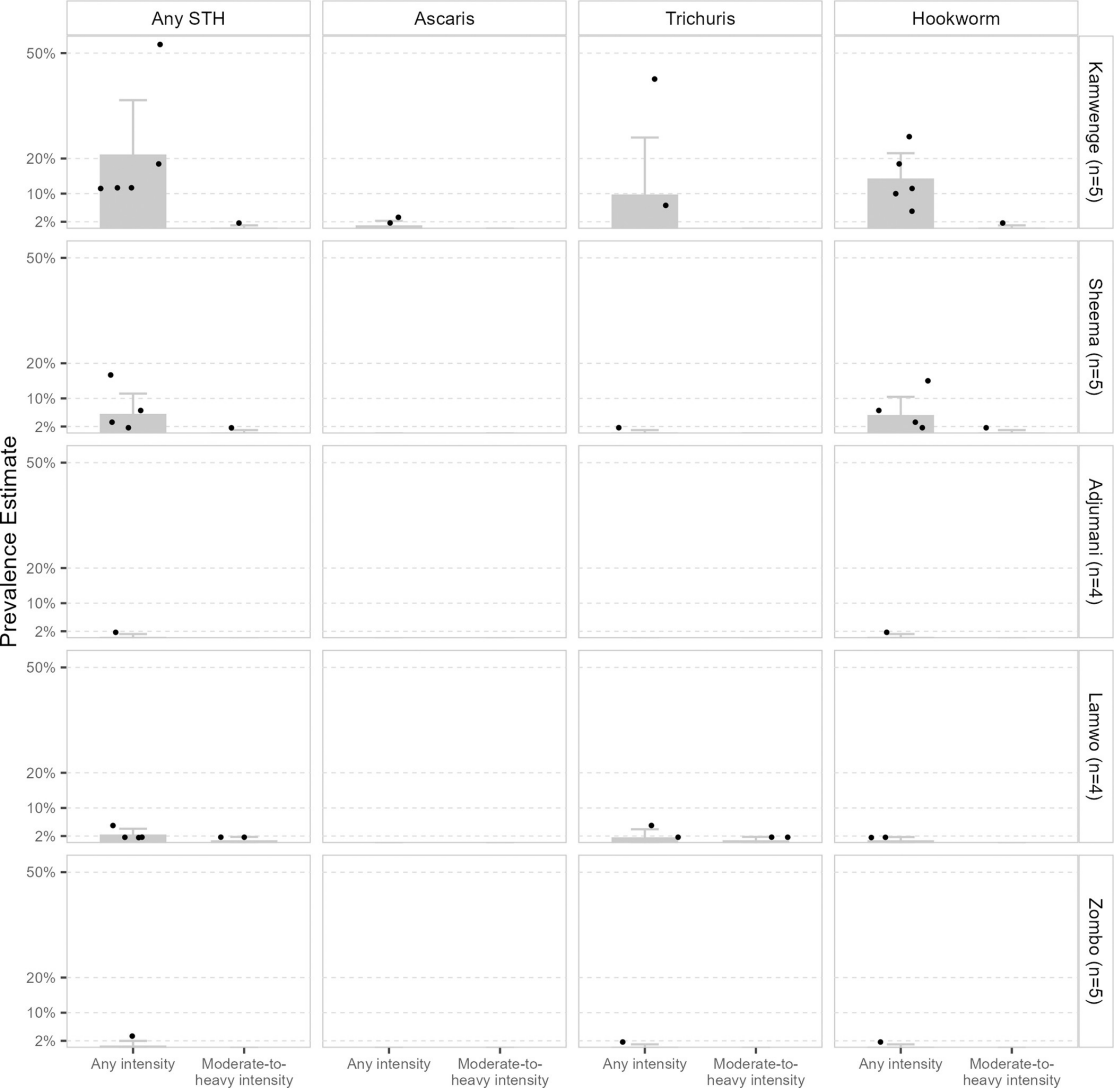
Estimated prevalence of soil-transmitted helminth infections by intensity, species, school, and district. Abbreviations: STH–Soil-transmitted helminth; n–Number of schools surveyed. Gray bars represent the district-level prevalence. Error bars represent the upper 95% confidence limit. Black dots represent the school-level prevalence. Only estimates >0% are plotted. Horizontal reference bars (dashed gray lines) represent thresholds for the World Health Organization-recommended preventive chemotherapy distribution frequency (based on any STH infection prevalence) [23].

District-level prevalence estimates are presented in Table 2 and Fig 2. The western districts had the highest prevalence of any STH infection, with Kamwenge District exhibiting a prevalence of >20% and Sheema of >5%. In contrast, the prevalence of any STH infection in the northern districts was very low to low, ranging from 0.4% to 2.4%. Moderate-to-heavy intensity infection prevalence of any STH was less than 1% in all surveyed districts.

**Table 2:** Estimated prevalence of soil-transmitted helminth infections by intensity, species, and district.

		Any intensity infections		Moderate-to-heavy intensity infections	
Species/District	Number of students surveyed	Number of students infected	Prevalence (%, 95% confidence limits)	Number of students infected	Prevalence (%, 95% confidence limits)
**Any species**					
Kamwenge (n = 5)	307	65	21.2 (5.7, 36.6)	1	0.3 (0.0, 1.0)
Sheema (n = 5)	303	17	5.6 (0.0, 11.4)	1	0.3 (0.0, 1.0)
Adjumani (n = 4)	244	1	0.4 (0.0, 1.2)	0	0.0 (0.0, 0.0)
Lamwo (n = 4)	245	6	2.4 (0.8, 4.1)	2	0.8 (0.0, 1.8)
Zombo (n = 5)	299	2	0.7 (0.0, 2.0)	0	0.0 (0.0, 0.0)
***Ascaris lumbricoides***					
Kamwenge (n = 5)	307	3	1.0 (0.0, 2.3)	0	0.0 (0.0, 0.0)
Sheema (n = 5)	303	0	0.0 (0.0, 0.0)	0	0.0 (0.0, 0.0)
Adjumani (n = 4)	244	0	0.0 (0.0, 0.0)	0	0.0 (0.0, 0.0)
Lamwo (n = 4)	245	0	0.0 (0.0, 0.0)	0	0.0 (0.0, 0.0)
Zombo (n = 5)	299	0	0.0 (0.0, 0.0)	0	0.0 (0.0, 0.0)
***Trichuris trichiura***					
Kamwenge (n = 5)	307	30	9.8 (0.0, 26.0)	0	0.0 (0.0, 0.0)
Sheema (n = 5)	303	1	0.3 (0.0, 1.0)	0	0.0 (0.0, 0.0)
Adjumani (n = 4)	244	0	0.0 (0.0, 0.0)	0	0.0 (0.0, 0.0)
Lamwo (n = 4)	245	4	1.6 (0.0, 3.9)	2	0.8 (0.0, 1.8)
Zombo (n = 5)	299	1	0.3 (0.0, 1.0)	0	0.0 (0.0, 0.0)
Hookworm*					
Kamwenge (n = 5)	307	44	14.3 (7.2, 21.5)	1	0.3 (0.0, 1.0)
Sheema (n = 5)	303	16	5.3 (0.1, 10.4)	1	0.3 (0.0, 1.0)
Adjumani (n = 4)	244	1	0.4 (0.0, 1.2)	0	0.0 (0.0, 0.0)
Lamwo (n = 4)	245	2	0.8 (0.0, 1.7)	0	0.0 (0.0, 0.0)
Zombo (n = 5)	299	1	0.3 (0.0, 1.0)	0	0.0 (0.0, 0.0)

Abbreviation: n–number of schools surveyed

*Ancylostoma duodenale and *Necator americanus*

School-level prevalence estimates are shown in Fig 2 and S1 Table. Within the northern districts, estimates generally remained consistently low within each district. However, heterogeneity was observed in Kamwenge District, where four schools showed moderate prevalence (10–<20%) of any STH infection, and one school recorded over 50% prevalence, driven by high *T*. *trichiura* and hookworm prevalence.

### Infections by species

*A*. *lumbricoides* infections were infrequent, with only three infections identified in two schools in the Kamwenge District. *T*. *trichiura* was generally uncommon, with prevalences ranging from 0.0% to 1.6% within all districts except Kamwenge. In Kamwenge District, roughly 10% of students were infected. However, one school accounted for approximately 85% of identified *T*. *trichiura* infections. Hookworm infections were predominantly found in the Western region districts of Kamwenge and Sheema, with moderate and low prevalence, respectively. All northern districts exhibited a <1% hookworm prevalence.

### Co-infections

**Fig 3:**
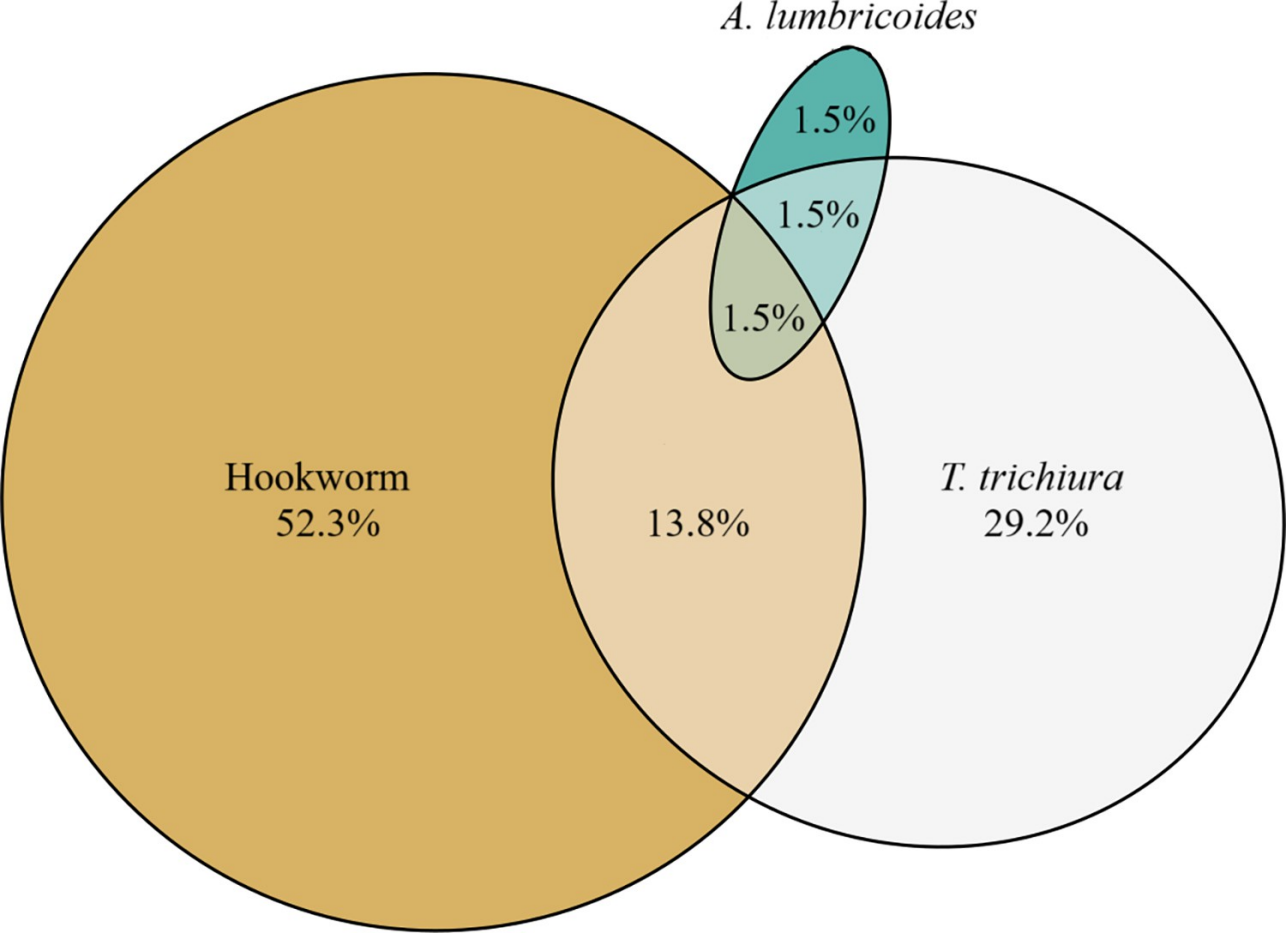
Co-infection status by species in Kamwenge District. Figure created at: https://eulerr.co/. The text has been modified.

Coinfections were not observed in Sheema, Adjumani, Lamwo, and Zombo Districts. In Kamwenge District, of the 65 identified infections, most were mono-infections (52.3% hookworm, 29.2% *T*. *trichiura*, and 1.5% *A*. *lumbricoides*, Fig 3). Coinfections were seen in 16.9% of the cases, with the primary combination being hookworm and *T*. *trichiura*. One triple infection was identified.

## Discussion

Results from school-based surveys conducted in five districts revealed a substantial decrease in STH infection prevalence compared to earlier national surveys. We found that the prevalence in the Sheema, Adjumani, Lamwo, and Zombo Districts was low (<6%), with correspondingly low moderate-to-heavy intensity infection prevalence (<1%). A reduction in PC frequency per WHO recommendations [23] may be warranted in these districts. However, the prevalence of any intensity infection remained over 20% in the Kamwenge District despite over a decade of deworming. This high prevalence can be partially attributed to one school with an estimated prevalence of >50%, suggesting intra-district heterogeneity and local factors contributing to STH transmission [14,24]. Despite the high prevalence of any-intensity infections in Kamwenge District, moderate-to-heavy-intensity infection prevalence was low, consistent with that observed in the other four surveyed districts.

The low moderate-to-heavy intensity infection prevalence estimates (<1%) in these five districts suggest that areas of Uganda are achieving the WHO 2030 Road Map target of the elimination of STH as a public health problem (defined as <2% moderate-to-heavy intensity infection prevalence among children) [14,23].

### Parasite species

The near absence of *A*. *lumbricoides* infections seen across the surveyed districts was a positive finding and aligned with expectations based on the high efficacy of benzimidazoles in treating these parasites [25].

However, both *T*. *trichiura* and hookworm infections persisted. Hookworm infections were most commonly observed in the western districts. Yet at least one infection was identified in each district, indicating a ubiquity consistent with baseline surveys conducted in Uganda [13,14]. Our surveys suggested hookworm control efforts have been more effective in the Northern region. This may be partially explained by the fact that the Northern region is co-endemic with onchocerciasis and has benefited from semi-annual mass drug administration with ivermectin, a treatment known to improve control of parasitic worms [26]. To address the persistent hookworm infections and the increased burden of podoconiosis in the western districts [27–29], intensified health education aimed at minimizing soil contact could significantly contribute to the control of these diseases.

The prevalence of *T*. *trichiura* was highest in the Kamwenge District and was driven by infections identified in only two of the five schools surveyed, with prevalences of 42.6% and 6.7%. One potential explanation for these elevated prevalences is the comparatively lower efficacy of mebendazole and albendazole against *T*. *trichiura* than other helminth species [25,26]. This reduced efficacy, coupled with the potential for drug resistance or programmatic shortcomings, underscores the need for comprehensive exploration into the underlying causes for the observed sub-optimal intervention response within these schools. Alternative treatments and enhanced control measures should be considered [2,3,30].

### Limitations

This survey has several limitations. First, two schools (one in Lamwo and one in Adjumani) were removed from the analysis, as records showed they distributed STH treatment within a month before this survey. However, a sensitivity analysis to explore the impact of these excluded schools (see S1 Text) indicated that the exclusion would have minimal impact on the PC treatment frequency category in these districts. Second, using a single stool sample, duplicate Kato Katz is suboptimal for detecting infections in low-prevalence settings [23]. However, this diagnostic approach is based on WHO-established indicators to direct PC treatment categories (prevalence of any intensity infections) and elimination as a public health problem (prevalence of moderate-to-heavy intensity infections). Hence, despite its limitations, we selected this method to align with the established standard. Third, hookworm eggs are susceptible to rapid degradation [31], especially in warm, humid climates. Although our laboratory facilities were located at the schools and samples were read shortly after deposit, with prepared slides examined within 60 minutes of preparation, egg degradation remains possible. However, due to our rigorous focus on maintaining sample integrity, we believe this poses only a minimal threat to the validity of our findings. Finally, we conducted school-based surveys to evaluate the prevalence among school-aged children. Non-school-attending children were not included in the survey population; therefore, when relating our findings to WHO-recommended PC treatment categories, we must assume that the prevalence among non-school-attending children is equivalent to that among school-attending children. Given the high school attendance in these districts and the availability of community-based distribution for PC, we consider this to be a minimal threat to our validity. Nonetheless, if non-school-attending children are less likely to receive deworming medications, our estimates may underestimate the true prevalence among school-aged children.

## Conclusions

Our results indicate significant progress in STH control, with all five districts surveyed exhibiting a moderate-to-heavy intensity infection prevalence below the 2% WHO threshold for eliminating STH infections as a public health problem. This achievement is likely attributed to Uganda’s extensive deworming program spanning two decades, which is believed to have substantially reduced STH-related morbidity among school-aged children. Nonetheless, despite the overall low prevalence of moderate-to-heavy intensity infections, the prevalence of any-intensity infections in one district remained high. This underscores the importance of sustained monitoring and vigilance to ensure that the 2030 targets can be achieved and maintained in all areas.

## Supporting information

S1 Text Sensitivity analysis for PC frequency categorization. (DOCX)

S1 Table School-level-results for each species. (DOCX)
