# Supplementary material for: Prevalence and intensity of soil-transmitted helminth infections among school-aged children in five districts in Uganda
Source: PLoS Negl Trop Dis. 2024 Aug 1;18(8):e0012324. doi: 10.1371/journal.pntd.0012324 (PMC11293726; doi:10.1371/journal.pntd.0012324)
Supplement: S1 Text — (DOCX) [file pntd.0012324.s001.docx]

Sensitivity analysis for PC frequency categorization

**Background**

The survey was designed to be conducted in five schools per district. However, it was later learned that one school in the Adjumani District and one in the Lamwo District had conducted deworming within the month before the surveys. Thus, these school's data were excluded, as prevalence is likely to be low due to recent deworming.

**Methods**

We conducted a sensitivity analysis to determine the impact of these school exclusions on the World Health Organization (WHO)-recommended preventive chemotherapy (PC) frequency categorization for the district (which is based on the district-level prevalence) [1]. We asked, “In each district, had 60 students from the excluded school contributed stool samples, what would the school-level prevalence needed to have been to move the district-level prevalence into the next PC frequency category?”

**Results**

In Adjumani District, the district-level prevalence of any STH infection among the four included schools was 0.4% (1/244), indicating a recommendation for PC suspension. To reach the next thresholds for PC frequency, assuming 60 students were hypothetically tested at the excluded school, the school would have had to have had a prevalence of 10.0% (6/60), 50.0% (30/60), and 100.0% (60/60) to reach the district-level PC treatment frequency thresholds of 2%, 10%, and 20%, respectively. Given that a school-level prevalence of ≥50% is unlikely, PC recommendations for suspension or once every two years are suggested by this sensitivity analysis.

In Lamwo District, the district-level prevalence of any STH without the excluded school was 2.4% (6/245), indicating a recommendation for PC once every two years. To reach the next thresholds for PC frequency, the excluded school would have had to have had a prevalence of 41.7% (25/60) and 91.7% (55/60) to reach the thresholds of 10% and 20%, respectively. Given that a school-level prevalence of ≥40% is unlikely, this suggests that the recommended PC treatment frequency for this district was unlikely to have been impacted by the school exclusion and that PC once every two years remains recommended.

**Reference**

1. Helminth control in school-age children: a guide for managers of control programmes, 2nd ed. Geneva: World Health Organization, 2011.
