# Supplementary material for: Prevalence and intensity of soil-transmitted helminth infections among school-aged children in five districts in Uganda
Source: PLoS Negl Trop Dis. 2024 Aug 1;18(8):e0012324. doi: 10.1371/journal.pntd.0012324 (PMC11293726; doi:10.1371/journal.pntd.0012324)
Supplement: S1 Table — (DOCX) [file pntd.0012324.s002.docx]

**Table a. Estimated prevalence of any soil-transmitted helminth infection by district, species, and intensity**

|  | **Number of 10–14-year-old students** | | **Any intensity  infections** | | **Moderate-to-heavy intensity infections** | |
| --- | --- | --- | --- | --- | --- | --- |
|  | **Enrolled*** | **Surveyed** | **Number infected** | **Prevalence  (%, 95% confidence limits)** | **Number infected** | **Prevalence  (%, 95% confidence limits)** |
| **Kamwenge (n=5)** | **1,942** | **307** | **65** | **21.2 (5.7, 36.6)** | **1** | **0.3 (0.0, 1.0)** |
| School 1 | 160 | 60 | 7 | 11.7 (3.5, 19.9) | 0 | 0.0 (0.0, 0.0) |
| School 2 | 126 | 60 | 7 | 11.7 (3.5, 19.9) | 0 | 0.0 (0.0, 0.0) |
| School 3 | 950 | 61 | 32 | 52.5 (39.8, 65.1) | 0 | 0.0 (0.0, 0.0) |
| School 4 | 283 | 61 | 7 | 11.5 (3.4, 19.5) | 1 | 1.6 (0.0, 4.9) |
| School 5 | 423 | 65 | 12 | 18.5 (9.0, 28.0) | 0 | 0.0 (0.0, 0.0) |
| **Sheema (n=5)** | **991** | **303** | **17** | **5.6 (0.0, 11.4)** | **1** | **0.3 (0.0, 1.0)** |
| School 1 | 209 | 62 | 2 | 3.2 (0.0, 7.7) | 0 | 0.0 (0.0, 0.0) |
| School 2 | 72 | 60 | 0 | 0.0 (0.0, 0.0) | 0 | 0.0 (0.0, 0.0) |
| School 3 | 190 | 61 | 4 | 6.6 (0.3, 12.8) | 1 | 1.6 (0.0, 4.9) |
| School 4 | 405 | 60 | 10 | 16.7 (7.2, 26.2) | 0 | 0.0 (0.0, 0.0) |
| School 5 | 115 | 60 | 1 | 1.7 (0.0, 4.9) | 0 | 0.0 (0.0, 0.0) |
| **Adjumani (n=4)** | **864** | **244** | **1** | **0.4 (0.0, 1.2)** | **0** | **0.0 (0.0, 0.0)** |
| School 1 | 188 | 59 | 1 | 1.7 (0.0, 5.0) | 0 | 0.0 (0.0, 0.0) |
| School 2 | 257 | 63 | 0 | 0.0 (0.0, 0.0) | 0 | 0.0 (0.0, 0.0) |
| School 3 | 163 | 61 | 0 | 0.0 (0.0, 0.0) | 0 | 0.0 (0.0, 0.0) |
| School 4 | 256 | 61 | 0 | 0.0 (0.0, 0.0) | 0 | 0.0 (0.0, 0.0) |
| **Lamwo (n=4)** | **876** | **245** | **6** | **2.4 (0.8, 4.1)** | **2** | **0.8 (0.0, 1.8)** |
| School 1 | 249 | 60 | 3 | 5.0 (0.0, 10.6) | 1 | 1.7 (0.0, 4.9) |
| School 2 | 223 | 61 | 1 | 1.6 (0.0, 4.9) | 0 | 0.0 (0.0, 0.0) |
| School 3 | 177 | 64 | 1 | 1.6 (0.0, 4.6) | 0 | 0.0 (0.0, 0.0) |
| School 4 | 227 | 60 | 1 | 1.7 (0.0, 4.9) | 1 | 1.7 (0.0, 4.9) |
| **Zombo (n=5)** | **1,673** | **299** | **2** | **0.7 (0.0, 2.0)** | **0** | **0.0 (0.0, 0.0)** |
| School 1 | 393 | 58 | 0 | 0.0 (0.0, 0.0) | 0 | 0.0 (0.0, 0.0) |
| School 2 | 276 | 60 | 0 | 0.0 (0.0, 0.0) | 0 | 0.0 (0.0, 0.0) |
| School 3 | 310 | 60 | 2 | 3.3 (0.0, 7.9) | 0 | 0.0 (0.0, 0.0) |
| School 4 | 198 | 61 | 0 | 0.0 (0.0, 0.0) | 0 | 0.0 (0.0, 0.0) |
| School 5 | 496 | 60 | 0 | 0.0 (0.0, 0.0) | 0 | 0.0 (0.0, 0.0) |

*Abbreviation: n – number of schools surveyed*

**Per enrollment figures provided to survey team prior to the survey*

**Table b. Estimated prevalence of *Ascaris lumbricoides* infections by district, species, and intensity**

|  | **Number of 10–14-year-old students** | | **Any intensity  infections** | | **Moderate-to-heavy intensity infections** | |
| --- | --- | --- | --- | --- | --- | --- |
|  | **Enrolled*** | **Surveyed** | **Number of students infected** | **Prevalence  (%, 95% confidence limits)** | **Number of students infected** | **Prevalence  (%, 95% confidence limits)** |
| **Kamwenge (n=5)** | **1,942** | **307** | **3** | **1.0 (0.0, 2.3)** | **0** | **0.0 (0.0, 0.0)** |
| School 1 | 160 | 60 | 0 | 0.0 (0.0, 0.0) | 0 | 0.0 (0.0, 0.0) |
| School 2 | 126 | 60 | 1 | 1.7 (0.0, 4.9) | 0 | 0.0 (0.0, 0.0) |
| School 3 | 950 | 61 | 2 | 3.3 (0.0, 7.8) | 0 | 0.0 (0.0, 0.0) |
| School 4 | 283 | 61 | 0 | 0.0 (0.0, 0.0) | 0 | 0.0 (0.0, 0.0) |
| School 5 | 423 | 65 | 0 | 0.0 (0.0, 0.0) | 0 | 0.0 (0.0, 0.0) |
| **Sheema (n=5)** | **991** | **303** | **0** | **0.0 (0.0, 0.0)** | **0** | **0.0 (0.0, 0.0)** |
| School 1 | 209 | 62 | 0 | 0.0 (0.0, 0.0) | 0 | 0.0 (0.0, 0.0) |
| School 2 | 72 | 60 | 0 | 0.0 (0.0, 0.0) | 0 | 0.0 (0.0, 0.0) |
| School 3 | 190 | 61 | 0 | 0.0 (0.0, 0.0) | 0 | 0.0 (0.0, 0.0) |
| School 4 | 405 | 60 | 0 | 0.0 (0.0, 0.0) | 0 | 0.0 (0.0, 0.0) |
| School 5 | 115 | 60 | 0 | 0.0 (0.0, 0.0) | 0 | 0.0 (0.0, 0.0) |
| **Adjumani (n=4)** | **864** | **244** | **0** | **0.0 (0.0, 0.0)** | **0** | **0.0 (0.0, 0.0)** |
| School 1 | 188 | 59 | 0 | 0.0 (0.0, 0.0) | 0 | 0.0 (0.0, 0.0) |
| School 2 | 257 | 63 | 0 | 0.0 (0.0, 0.0) | 0 | 0.0 (0.0, 0.0) |
| School 3 | 163 | 61 | 0 | 0.0 (0.0, 0.0) | 0 | 0.0 (0.0, 0.0) |
| School 4 | 256 | 61 | 0 | 0.0 (0.0, 0.0) | 0 | 0.0 (0.0, 0.0) |
| **Lamwo (n=4)** | **876** | **245** | **0** | **0.0 (0.0, 0.0)** | **0** | **0.0 (0.0, 0.0)** |
| School 1 | 249 | 60 | 0 | 0.0 (0.0, 0.0) | 0 | 0.0 (0.0, 0.0) |
| School 2 | 223 | 61 | 0 | 0.0 (0.0, 0.0) | 0 | 0.0 (0.0, 0.0) |
| School 3 | 177 | 64 | 0 | 0.0 (0.0, 0.0) | 0 | 0.0 (0.0, 0.0) |
| School 4 | 227 | 60 | 0 | 0.0 (0.0, 0.0) | 0 | 0.0 (0.0, 0.0) |
| **Zombo (n=5)** | **1,673** | **299** | **0** | **0.0 (0.0, 0.0)** | **0** | **0.0 (0.0, 0.0)** |
| School 1 | 393 | 58 | 0 | 0.0 (0.0, 0.0) | 0 | 0.0 (0.0, 0.0) |
| School 2 | 276 | 60 | 0 | 0.0 (0.0, 0.0) | 0 | 0.0 (0.0, 0.0) |
| School 3 | 310 | 60 | 0 | 0.0 (0.0, 0.0) | 0 | 0.0 (0.0, 0.0) |
| School 4 | 198 | 61 | 0 | 0.0 (0.0, 0.0) | 0 | 0.0 (0.0, 0.0) |
| School 5 | 496 | 60 | 0 | 0.0 (0.0, 0.0) | 0 | 0.0 (0.0, 0.0) |

*Abbreviation: n – number of schools surveyed*

**Per enrollment figures provided to survey team prior to the survey*

**Table c. Estimated prevalence of *Trichuris trichiura* infections by district, species, and intensity**

|  | **Number of 10–14-year-old students** | | **Any intensity**  **infections** | | **Moderate-to-heavy intensity infections** | |
| --- | --- | --- | --- | --- | --- | --- |
|  | **Enrolled*** | **Surveyed** | **Number of students infected** | **Prevalence  (%, 95% confidence limits)** | **Number of students infected** | **Prevalence  (%, 95% confidence limits)** |
| **Kamwenge (n=5)** | **1,942** | **307** | **30** | **9.8 (0.0, 26.0)** | **0** | **0.0 (0.0, 0.0)** |
| School 1 | 160 | 60 | 4 | 6.7 (0.3, 13.0) | 0 | 0.0 (0.0, 0.0) |
| School 2 | 126 | 60 | 0 | 0.0 (0.0, 0.0) | 0 | 0.0 (0.0, 0.0) |
| School 3 | 950 | 61 | 26 | 42.6 (30.1, 55.1) | 0 | 0.0 (0.0, 0.0) |
| School 4 | 283 | 61 | 0 | 0.0 (0.0, 0.0) | 0 | 0.0 (0.0, 0.0) |
| School 5 | 423 | 65 | 0 | 0.0 (0.0, 0.0) | 0 | 0.0 (0.0, 0.0) |
| **Sheema (n=5)** | **991** | **303** | **1** | **0.3 (0.0, 1.0)** | **0** | **0.0 (0.0, 0.0)** |
| School 1 | 209 | 62 | 0 | 0.0 (0.0, 0.0) | 0 | 0.0 (0.0, 0.0) |
| School 2 | 72 | 60 | 0 | 0.0 (0.0, 0.0) | 0 | 0.0 (0.0, 0.0) |
| School 3 | 190 | 61 | 0 | 0.0 (0.0, 0.0) | 0 | 0.0 (0.0, 0.0) |
| School 4 | 405 | 60 | 1 | 1.7 (0.0, 4.9) | 0 | 0.0 (0.0, 0.0) |
| School 5 | 115 | 60 | 0 | 0.0 (0.0, 0.0) | 0 | 0.0 (0.0, 0.0) |
| **Adjumani (n=4)** | **864** | **244** | **0** | **0.0 (0.0, 0.0)** | **0** | **0.0 (0.0, 0.0)** |
| School 1 | 188 | 59 | 0 | 0.0 (0.0, 0.0) | 0 | 0.0 (0.0, 0.0) |
| School 2 | 257 | 63 | 0 | 0.0 (0.0, 0.0) | 0 | 0.0 (0.0, 0.0) |
| School 3 | 163 | 61 | 0 | 0.0 (0.0, 0.0) | 0 | 0.0 (0.0, 0.0) |
| School 4 | 256 | 61 | 0 | 0.0 (0.0, 0.0) | 0 | 0.0 (0.0, 0.0) |
| **Lamwo (n=4)** | **876** | **245** | **4** | **1.6 (0.0, 3.9)** | **2** | **0.8 (0.0, 1.8)** |
| School 1 | 249 | 60 | 3 | 5.0 (0.0, 10.6) | 1 | 1.7 (0.0, 4.9) |
| School 2 | 223 | 61 | 0 | 0.0 (0.0, 0.0) | 0 | 0.0 (0.0, 0.0) |
| School 3 | 177 | 64 | 0 | 0.0 (0.0, 0.0) | 0 | 0.0 (0.0, 0.0) |
| School 4 | 227 | 60 | 1 | 1.7 (0.0, 4.9) | 1 | 1.7 (0.0, 4.9) |
| **Zombo (n=5)** | **1,673** | **299** | **1** | **0.3 (0.0, 1.0)** | **0** | **0.0 (0.0, 0.0)** |
| School 1 | 393 | 58 | 0 | 0.0 (0.0, 0.0) | 0 | 0.0 (0.0, 0.0) |
| School 2 | 276 | 60 | 0 | 0.0 (0.0, 0.0) | 0 | 0.0 (0.0, 0.0) |
| School 3 | 310 | 60 | 1 | 1.7 (0.0, 4.9) | 0 | 0.0 (0.0, 0.0) |
| School 4 | 198 | 61 | 0 | 0.0 (0.0, 0.0) | 0 | 0.0 (0.0, 0.0) |
| School 5 | 496 | 60 | 0 | 0.0 (0.0, 0.0) | 0 | 0.0 (0.0, 0.0) |

*Abbreviation: n – number of schools surveyed*

**Per enrollment figures provided to survey team prior to the survey*

**Table d. Estimated prevalence of hookworm* infections by district, species, and intensity**

| **Hookworm** | **Number of 10–14-year-old students** | | **Any intensity**  **infections** | | **Moderate-to-heavy intensity infections** | |
| --- | --- | --- | --- | --- | --- | --- |
|  | **Enrolled***^†^* | **Surveyed** | **Number of students infected** | **Prevalence  (%, 95%  confidence limits)** | **Number of students infected** | **Prevalence  (%, 95%  confidence limits)** |
| **Kamwenge (n=5)** | **1,942** | **307** | **44** | **14.3 (7.2, 21.5)** | **1** | **0.3 (0.0, 1.0)** |
| School 1 | 160 | 60 | 3 | 5.0 (0.0, 10.6) | 0 | 0.0 (0.0, 0.0) |
| School 2 | 126 | 60 | 6 | 10.0 (2.3, 17.7) | 0 | 0.0 (0.0, 0.0) |
| School 3 | 950 | 61 | 16 | 26.2 (15.1, 37.4) | 0 | 0.0 (0.0, 0.0) |
| School 4 | 283 | 61 | 7 | 11.5 (3.4, 19.5) | 1 | 1.6 (0.0, 4.9) |
| School 5 | 423 | 65 | 12 | 18.5 (9.0, 28.0) | 0 | 0.0 (0.0, 0.0) |
| **Sheema (n=5)** | **991** | **303** | **16** | **5.3 (0.1, 10.4)** | **1** | **0.3 (0.0, 1.0)** |
| School 1 | 209 | 62 | 2 | 3.2 (0.0, 7.7) | 0 | 0.0 (0.0, 0.0) |
| School 2 | 72 | 60 | 0 | 0.0 (0.0, 0.0) | 0 | 0.0 (0.0, 0.0) |
| School 3 | 190 | 61 | 4 | 6.6 (0.3, 12.8) | 1 | 1.6 (0.0, 4.9) |
| School 4 | 405 | 60 | 9 | 15.0 (5.9, 24.1) | 0 | 0.0 (0.0, 0.0) |
| School 5 | 115 | 60 | 1 | 1.7 (0.0, 4.9) | 0 | 0.0 (0.0, 0.0) |
| **Adjumani (n=4)** | **864** | **244** | **1** | **0.4 (0.0, 1.2)** | **0** | **0.0 (0.0, 0.0)** |
| School 1 | 188 | 59 | 1 | 1.7 (0.0, 5.0) | 0 | 0.0 (0.0, 0.0) |
| School 2 | 257 | 63 | 0 | 0.0 (0.0, 0.0) | 0 | 0.0 (0.0, 0.0) |
| School 3 | 163 | 61 | 0 | 0.0 (0.0, 0.0) | 0 | 0.0 (0.0, 0.0) |
| School 4 | 256 | 61 | 0 | 0.0 (0.0, 0.0) | 0 | 0.0 (0.0, 0.0) |
| **Lamwo (n=4)** | **876** | **245** | **2** | **0.8 (0.0, 1.7)** | **0** | **0.0 (0.0, 0.0)** |
| School 1 | 249 | 60 | 0 | 0.0 (0.0, 0.0) | 0 | 0.0 (0.0, 0.0) |
| School 2 | 223 | 61 | 1 | 1.6 (0.0, 4.9) | 0 | 0.0 (0.0, 0.0) |
| School 3 | 177 | 64 | 1 | 1.6 (0.0, 4.6) | 0 | 0.0 (0.0, 0.0) |
| School 4 | 227 | 60 | 0 | 0.0 (0.0, 0.0) | 0 | 0.0 (0.0, 0.0) |
| **Zombo (n=5)** | **1,673** | **299** | **1** | **0.3 (0.0, 1.0)** | **0** | **0.0 (0.0, 0.0)** |
| School 1 | 393 | 58 | 0 | 0.0 (0.0, 0.0) | 0 | 0.0 (0.0, 0.0) |
| School 2 | 276 | 60 | 0 | 0.0 (0.0, 0.0) | 0 | 0.0 (0.0, 0.0) |
| School 3 | 310 | 60 | 1 | 1.7 (0.0, 4.9) | 0 | 0.0 (0.0, 0.0) |
| School 4 | 198 | 61 | 0 | 0.0 (0.0, 0.0) | 0 | 0.0 (0.0, 0.0) |
| School 5 | 496 | 60 | 0 | 0.0 (0.0, 0.0) | 0 | 0.0 (0.0, 0.0) |

*Abbreviation: n – number of schools surveyed*

**Ancylostoma duodenale and Necator americanus*

*^†^Per enrollment figures provided to survey team prior to the survey*
